# Integrated photonics multi-waveguide devices for optical trapping and Raman spectroscopy: design, fabrication and performance demonstration

**DOI:** 10.3762/bjnano.11.68

**Published:** 2020-05-27

**Authors:** Gyllion B Loozen, Arnica Karuna, Mohammad M R Fanood, Erik Schreuder, Jacob Caro

**Affiliations:** 1 Department of Imaging Physics, Delft University of Technology, Lorentzweg 1, 2628 CJ Delft, Netherlands; 2 present address: Institute of Physical Chemistry, Friedrich Schiller University Jena, 07737 Jena, Germany; 3 LioniX International B.V., P.O. Box 456, 7500 AL, Enschede, Netherlands

**Keywords:** Brownian motion, integrated optics devices, lab-on-a-chip, optical trapping, nanofabrication, Raman spectroscopy, ridge waveguides

## Abstract

We realized integrated photonics multi-waveguide devices for optical trapping and Raman spectroscopy of particles in a fluid. In these devices, multiple beams directed towards the device center lead to a local field enhancement around this center and thus counteract the effect of light concentration near the facets, which is a disadvantage of dual-waveguide traps. Thus, a trapping region is created around the center, where a single particle of a size in a wide range can be trapped and studied spectroscopically, free from the influence of surfaces. We report the design (including simulations), fabrication and performance demonstration for multi-waveguide devices, using our Si_3_N_4_ waveguiding platform as the basis. The designed ridge waveguides, optimized for trapping and Raman spectroscopy, emit narrow beams. Multiple waveguides arranged around the central microbath result from fanning out of a single input waveguide using Y-splitters. A second waveguiding layer is implemented for detection of light scattered by the trapped particle. For reliable filling of the device with sample fluid, microfluidic considerations lead to side channels of the microbath, to exploit capillary forces. The interference of the multiple beams produces an array of hot spots around the bath center, each forming a local trap. This property is clearly confirmed in the experiments and is registered in videos. We demonstrate the performance of a 2-waveguide and a 16-waveguide device, using 1 and 3 μm polystyrene beads. Study of the confined Brownian motion of the trapped beads yields experimental values of the normalized trap stiffness for the in-plane directions. The stiffness values for the 16-waveguide device are comparable to those of tightly focused Gaussian beam traps and are confirmed by our own simulations. The Raman spectra of the beads (in this work measured via an objective) show clear peaks that are characteristic of polystyrene. In the low-wavenumber range, the spectra have a background that most likely originates from the Si_3_N_4_ waveguides.

## Introduction

Photonic lab-on-a-chip (LOC) techniques strongly attract attention for the manipulation and measurement of biological particles such as bacteria and various types of biological cells [1]. In this context, LOC devices for optical trapping and Raman spectroscopy are very promising. An ultimate goal for such LOC devices is on-the-spot identification of single biological particles by Raman spectroscopy using a chip-based portable system. These LOC devices are on-chip versions of a laser-tweezers Raman spectroscopy (LTRS) setup, which is a free space optics instrument. In LTRS, optical trapping and Raman spectroscopy of a particle are carried out using a focused laser beam, enabling the label-free analysis of single cells in an aqueous suspension away from surfaces [2]. For on-chip trapping and Raman spectroscopy, the dual-beam trap based on fibers or integrated photonics waveguides has been studied extensively [3–6]. This trap comprises two excitation fibers or excitation waveguides, which emit counter-propagating beams into a fluidic environment, which is often a fluidic channel for particle delivery. The beams interfere and create a volume of highly concentrated light that is suitable for optical trapping and Raman spectroscopy.

In [3], for example, a dual-fiber trap is used to trap tumor cells and blood cells, while Raman spectra are induced and collected by an external spectroscopy system. This work was extended in [4] by using fibers for both trapping of single polystyrene beads and for inducing and collecting Raman signals for the trapped beads. In our previous work [5], we used integrated photonics Si_3_N_4_ waveguides of a box shape and demonstrated for polystyrene beads the simultaneous optical trapping and Raman excitation of polystyrene beads using the same counter-propagating beams. The important advantages of integrated photonic waveguides are the high degree of control in fabrication (down to the nanometer scale) and the mass producibility. In [6], we presented a detailed simulation study of the trapping capabilities for extracellular vesicles (EVs) of the dual-waveguide trap we used in [5]. EVs are small cell-derived particles (diameter ranging from 30 to 1000 nm) and are important as potential biomarkers for cancer. In [6] we found, due to the divergence of the emitted beams, that larger facet separations (≥10 μm) lead to a strong global hot region of the light field near the waveguide facets. These global hot regions are preferential trapping sites, which may lead to adherence of the particle to the facets and disturbance of its Raman spectrum due to particle–surface interaction, which are effects to be avoided. It is of interest to compare the devices described in [3–6] with the long hollow core fibers for fiber-enhanced Raman spectroscopy used in [7]. With the technique applied in [7], owing to the long sample length, strong Raman signals of an ensemble of particles can be measured for small sample volumes. This is in contrast with the techniques used in [3–6], which enable measuring the Raman spectrum of a single particle, i.e., not an ensemble average, and thus enable detecting differences between individual particles of the same type that sequentially enter the active device part.

Here, we solve the problem of a global hot region near the facets by realizing *multi*-waveguide devices for trapping and Raman spectroscopy. In these devices, multiple nanofabricated Si_3_N_4_ excitation waveguides launch multiple coherent beams towards the center of the device, leading to a field enhancement around the center and thus effectively neutralizing the light concentration near the facets. In this way, a region of trapping is realized around the device center, where a single particle of a size in a wide range can be studied free from surfaces, while being trapped in the aqueous medium. This concept of light concentration in the device center was first proposed and realized in [8], but in that work, optical fibers instead of nanofabricated waveguides for beam emission were used, manually glued between two V-grooves of a holder.

**Figure 1:**
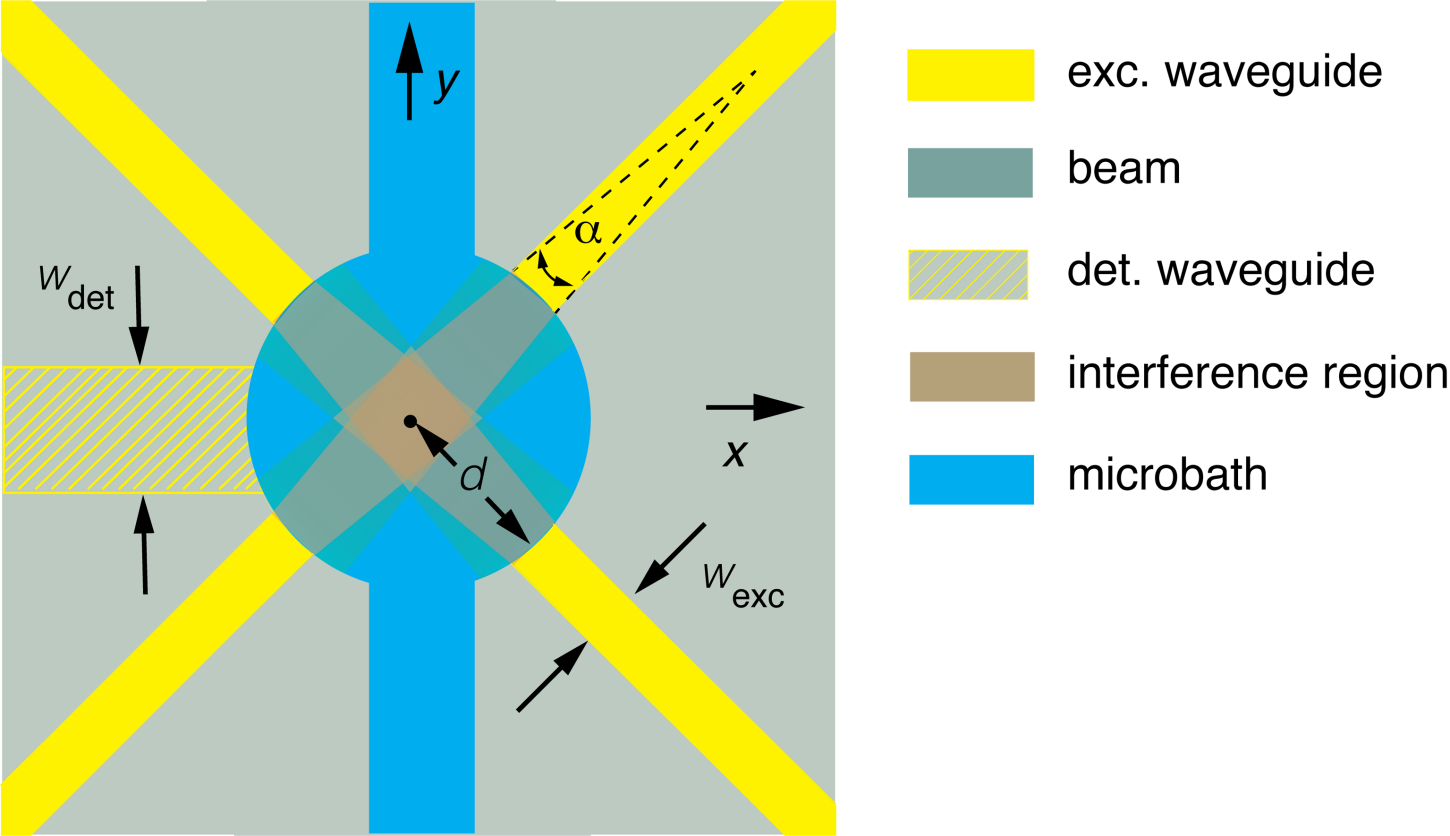
Schematic of the device concept with four excitation waveguides. A single detection waveguide of a set of several is indicated as well. The cylindrical microbath has two side channels. Beams emitted from the excitation waveguides into the microbath overlap in the central region, where interference and field enhancement occur. The direction of the Cartesian coordinates *x* and *y*, with the origin placed in the microbath center, is indicated. *w*_exc_ is the excitation-waveguide width, *w*_det_ is the detection-waveguide width, α is the angle of divergence of the emitted beam and *d* is the distance from the waveguide facet to the microbath.

In Figure 1 we present a schematic drawing of a 4-waveguide device, in order to explain the concept we apply. Four excitation waveguides are arranged around a cylindrical fluidic microbath that can be filled in with a suspension of particles. Detection waveguides, located in another waveguiding layer, are arranged similarly (only a single detection waveguide is shown in Figure 1, as an example). Several quantities characterize a multi-waveguide device and determine its performance. These are (see Figure 1) the widths *w*_exc_ and *w*_det_ of the excitation and detection waveguides, respectively, the typical angle of divergence α of the emitted beam, the region of overlap of the multiple beams where interference occurs, and the distance *d* of the waveguide facets to the center of the overlap region (*d* also defines the size of the microbath). Apart from the width of a waveguide, also its thickness is an important parameter, both for the excitation and the detection waveguides. For the excitation waveguides, width and thickness together determine the quality of the emitted beam, i.e., its narrowness and thus the size of the three-dimensional region of overlap of the beams. Finally, the number of waveguides that emit beams is an important parameter as well.

This article is organized as follows. In the section on the design of the devices, we present the design of the excitation waveguides, the detection waveguides and the microbath. For the waveguides, we arrive at a specific choice based on the Si_3_N_4_ waveguiding platform that we have available. In the Experimental section, we present the fabrication process of the devices and describe the experimental setup and the preparation of the sample. In the Results and Discussion section we demonstrate the performance of the multi-waveguide devices, both for trapping and Raman spectroscopy. In the demonstration we compare a 2-waveguide device with a 16-waveguide device, using polystyrene beads as test particles. Finally, we present the conclusions of our study.

## Design of the multi-waveguide devices for trapping and Raman spectroscopy

We carried out extensive design procedures for the multi-waveguide devices to arrive at optimum designs for their functional parts. These functional parts are described in the following (see Figure 1).

### The excitation waveguides and their circuitry

Here, we simulated the beam emitted by the waveguide as a function of the waveguide width, designed the connecting circuitry of the excitation waveguides, calculated the fiber-to-waveguide transmission, and finally, simulated the energy density in the microbath resulting from the multiple beams.

### The detection waveguides and their circuitry

In this case, the main design approach was to optimize the collection efficiency of the detection waveguides, which is determined by the area of their input facets and the distance to the microbath center.

### The microbath

The design of the microbath was guided by common microfluidic considerations, also making sure that the microbath is compatible with the geometry of the excitation waveguides.

### The excitation waveguides, their circuitry and their arrangement around the microbath

The light beams emitted by the multiple excitation waveguides should lead to a strong light concentration in the central region of the microbath. This implies that the beams should be narrow and have a low divergence. To realize this, we chose the single-stripe waveguide of our TripleX waveguiding platform [9]. This is a rectangular Si_3_N_4_ ridge waveguide embedded in SiO_2_ cladding. The TripleX platform offers high transparency across the wide wavelength range of 405–2350 nm, which includes our laser wavelength of 785 nm used for trapping and Raman spectroscopy. Single-stripe waveguides require considerably fewer fabrication steps than the box-shaped TripleX waveguides we used before [5–6]. This is the reason for our choice, where we take into account that the present devices, apart from the excitation waveguides, also have detection waveguides located in a separate waveguiding layer.

### Excitation waveguides

To determine the thickness of the excitation waveguides, we simulated the beam emitted from the facet into water (the typical medium in our experiments) for various waveguide thicknesses using the 3D finite-difference time-domain (FDTD) method with Lumerical’s FDTD solutions [10]. We choose a waveguide width *w*_exc_ of 1 μm, which is the minimum width for the contact lithography we use. We aim for single-mode operation of the waveguides at 785 nm for the transverse magnetic (TM) polarization. For TM polarization, the electric field vector 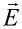 of the waveguide mode is directed perpendicular to the plane of the waveguide (the *x–y* plane, as indicated in Figure 1). This polarization is conserved in the emitted beam. For a multi-waveguide configuration as shown in Figure 1, the polarization of each beam then points in the same direction. For equal optical path lengths from the waveguide facets to the microbath center and for beams leaving the facets in phase, the light concentration in the center reaches the maximum obtainable value for the TM polarization, as a result of optimum constructive interference. For transverse electric (TE) polarization, for which the electric field vector 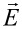 of the beams is oriented in the *x–y* plane, the resulting light concentration is lower.

**Figure 2:**
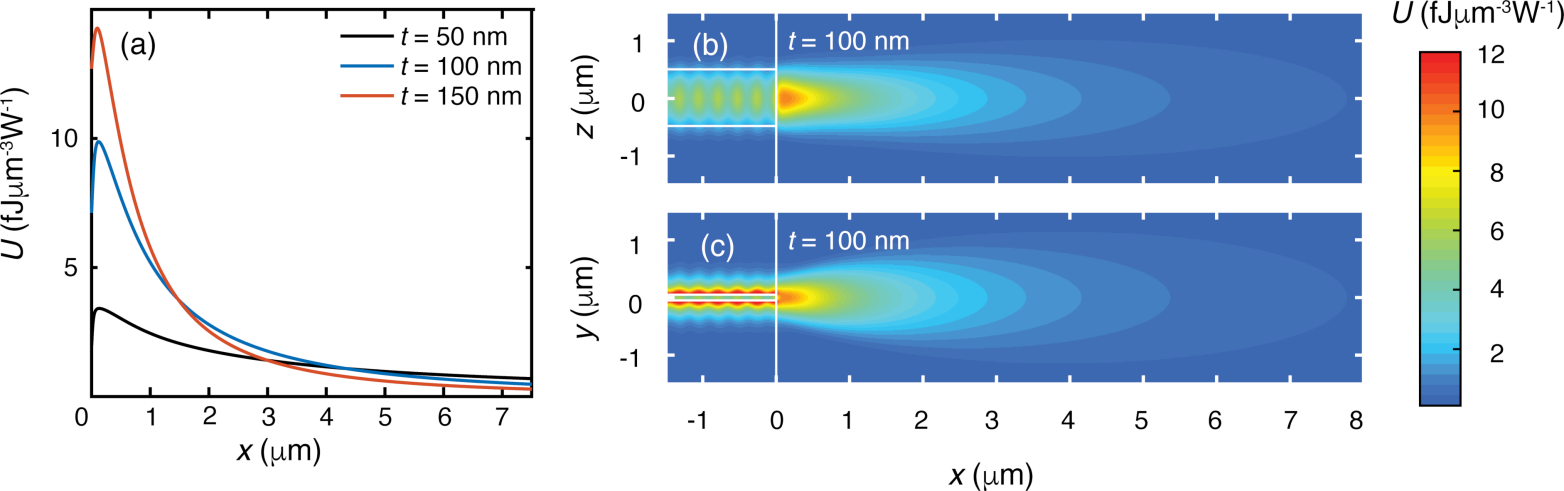
(a) Longitudinal profiles of the energy density *U* along the beam axis for waveguide thicknesses of 50, 100 and 150 nm, for 1 W of power delivered to the waveguide mode. The waveguide width is 1 μm. (b) and (c) show *U* of the beam emitted into water by the 100 nm thick waveguide in the *x*–*z* plane and the *x*–*y* plane, respectively. Color bar calibrated for 1 W of power delivered to the waveguide mode. The white vertical lines indicate the facet position, while the white horizontal lines indicate the waveguide. The periodic pattern inside and outside the waveguide in (b) and (c) results from the interference of the forward propagating mode and the mode partially backreflected at the nitride–water interface.

In the simulations, the refractive index of silicon nitride, silicon oxide and water is chosen as *n*_Si3N4_ = 2.00, *n*_SiO2_ = 1.45, and *n*_H20_ = 1.33, respectively. To obtain the characteristics of the emitted beams, we follow the simulation approach of our previous work [6]. Figure 2a shows the longitudinal profiles of the energy density *U* of the electric field (per watt of power delivered to the waveguide mode) of the beams emitted into water and for waveguide thicknesses *t* = 50, 100 and 150 nm. The *x*-axis is the axis of the waveguide. While for *t* = 50 nm the profile is flattest (and thus the least divergent), the profile for *t* = 100 nm has the highest energy density in the *x*-range of 1.5–4.5 μm. The latter property is advantageous for multiple waveguides around a microbath with a radius of about 3 μm (optimum for, say, 1 μm diameter particles and smaller), since the strongest field enhancement can be realized using multiple beams for such a microbath size. A thickness of *t* = 100 nm is also appropriate for larger microbaths (more suitable for particles larger than 1 μm), since larger particles require a lower concentration of light for trapping and Raman spectroscopy. We thus choose *t* = 100 nm. For this thickness, only a single TM mode can exist in the waveguide. In Figure 2b and Figure 2c, we show the energy density of the beam emitted by the 100 nm thick waveguide in the *x–y* and the *x–z* plane, respectively. The highest density occurs close to the facet, followed by a decay, which are features also seen in Figure 2a. The beam in the *x–z* plane shows less lateral spreading than in the *x–y* plane, indicating that the width of 1 μm is not limiting here in obtaining a narrow beam.

### Connecting circuitry of the excitation waveguides

For introducing light into the multiple excitation waveguides, we have designed connecting circuitry comprising waveguides of the same dimensions as the excitation waveguides. Starting from the chip edge, a single input waveguide (to which a fiber can be coupled) fans out using 50/50 Y-splitters into multiple waveguides, which connect to the excitation waveguides of the specific device design. This multi-waveguide circuitry across the chip is designed with a script-based editor of Synopsys (OptoDesigner) for efficient waveguide routing, guided by the symmetry of the configuration of the excitation waveguides. In this we impose a minimum waveguide-bend radius of 300 μm to avoid bend losses exceeding −0.01 dB·cm^−1^. The underlying relation of bend loss versus bend radius was obtained from simulations. The estimated scattering loss at each Y-splitter is −0.5 dB. The intrinsic waveguide propagation loss for the chosen width and thickness is −0.5 dB∙cm^−1^. For the waveguide lengths used, the intrinsic waveguide propagation loss is negligible compared to the losses just mentioned. Figure 6d below gives an impression of the connecting circuitry of a 16-waveguide device.

### Fiber-to-waveguide coupling

**Figure 3:**
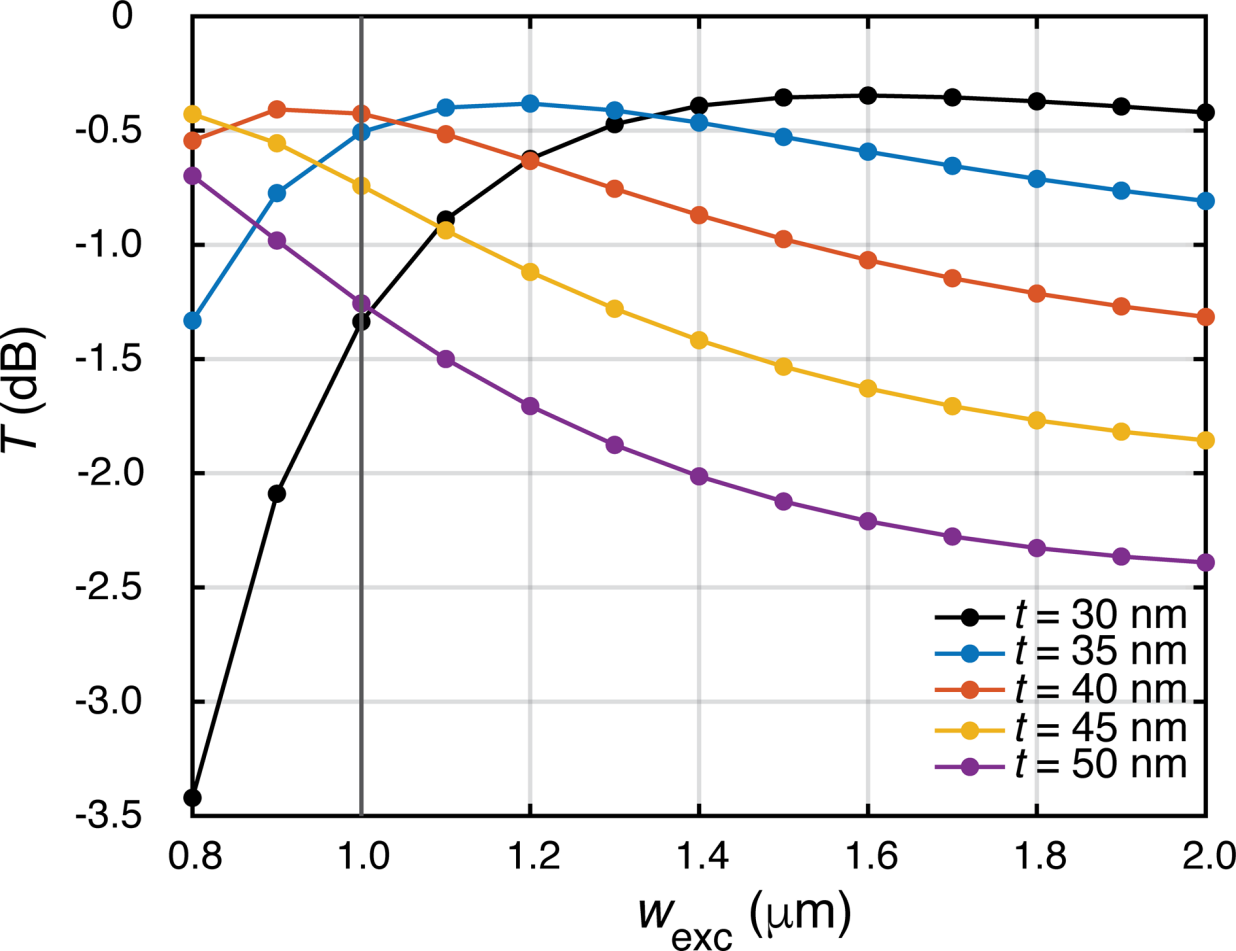
Fiber-to-chip transmission *T* as a function of the waveguide width *w*_exc_ for various thicknesses *t*. The vertical line indicates the chosen waveguide width.

To optimize the light coupling from a single-mode polarization maintaining fiber (a Thorlabs PM780-HP fiber, mode-field diameter of 5.3 μm) to the input waveguide at the chip edge, we numerically calculate the fiber-to-waveguide power transmission as a function of waveguide width and thickness. For this we use the overlap-integral expression for the electric fields of the fiber mode and the waveguide mode. The results are plotted in Figure 3. For *w*_exc_ = 1 μm, a thickness *t* between 35 and 40 nm yields optimum transmission of −0.5 dB or 89%. We further calculate the tolerance of the transmission against fabrication variability of the waveguide width and thickness, using thickness and width variations of ±5 nm and ±200 nm, respectively. This leads to the choice *t* = 35 nm at the chip edge. To obtain a thickness of 35 nm for the input waveguide at the chip edge, the waveguide is tapered down towards the chip edge (see subsection on fabrication below).

### Energy density in the microbath

**Figure 4:**
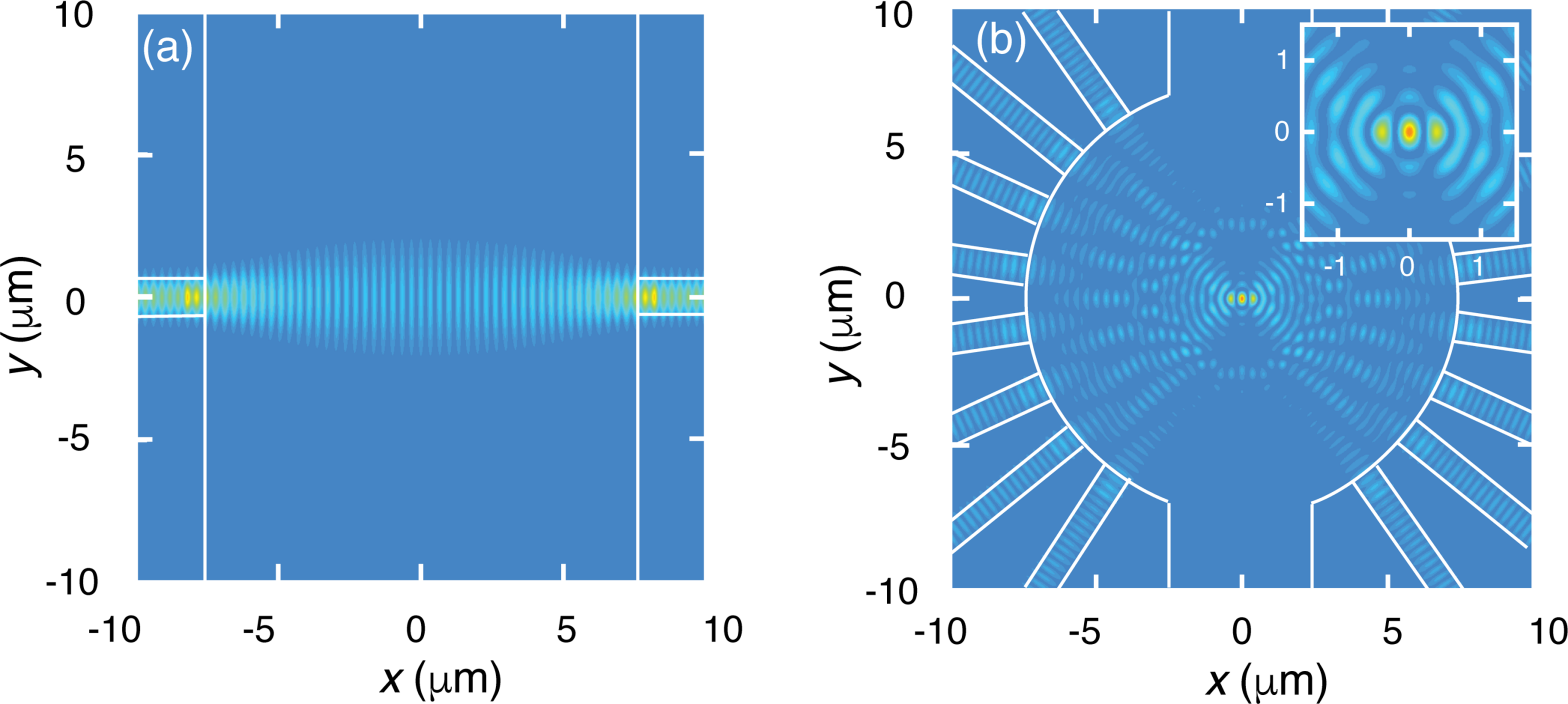
Energy density of the electric field in the multi-waveguide devices used in experimental demonstrations in this work. (a) 2-waveguide device with a 15 μm wide fluidic channel. (b) 16-waveguide device with a 15 μm diameter microbath and 5 μm wide side channels. The waveguides and the walls of the fluidic structures are indicated by white lines. For (a) and (b) the color scale indicating the energy density is the same.

We have designed various multi-waveguide devices with excitation waveguides of the type chosen above, with the number of waveguides varying between 2 and 32. In this work, we focus on experiments with a 2-waveguide and a 16-waveguide device. The 2-waveguide device has a linear, 15 μm wide fluidic channel with a rectangular cross section between the waveguides, while the 16-waveguide device has a cylindrical fluidic microbath with a diameter of 15 μm. Using Lumerical’s FDTD solutions, we obtain the energy density *U* in the central part of these devices, assuming the beams are emitted in phase. The results are presented in Figure 4. The 2-waveguide device (Figure 4a) shows a characteristic periodic pattern for *U*, with high values near the facets (global hot region). This pattern results from the interference of the emitted counter-propagating beams. The distance between the interference maxima is 785 nm/(2·*n*_H2O_) = 295 nm. Each interference maximum (local hot spot) is clearly narrower in the *x*-direction than in the *y*-direction. Particles can be trapped at the local hot spots. The global hot regions are preferential trapping regions. These may pose a problem for larger particles in view of possible adherence to the facet. For the 16-waveguide device, the interference pattern is completely different, as shown in Figure 4b. In this case there is a global hot region in the center of the microbath, resulting from the interference of the 16 beams, as intended. This is the preferential trapping region of this device. The structure of the global hot region is magnified in the inset of Figure 4b, showing that the hottest spot has two strong side lobes. Further outwards the lobes become increasingly weaker. The individual local hot spots at and near the center serve as local traps for small particles (≤295 nm, the typical distance between maxima of *U*), while larger particles are trapped as a result of the forces exerted by the multiple hot spots.

We note that real devices do not have in-phase beams due to unequal path lengths and phase errors accumulated by the guided modes. For the 2-waveguide device, this only gives a maximum shift of the interference pattern over a distance of half the period. For the 16-waveguide device, however, 16 random phases of arriving beams lead to a modified and weaker interference pattern with a random structure. This pattern of local hot spots will nevertheless lead to trapping effects similar to those for the ideal pattern resulting from in-phase beams.

### Detection waveguides and their circuitry

The Si_3_N_4_ detection waveguides are located in a separate waveguiding layer and serve to collect light scattered by the trapped particle, whether it is Raman scattered light or light of another origin. In the design, we optimize the efficiency of the waveguides to collect scattered light. The input facets of the waveguides are located at the circumference of the microbath, so as to realize maximum coverage of the circumference with multiple waveguides. We arrive at a maximum waveguide width *w*_det_ of 13.5 μm, the actual width varying among the devices. For the different microbaths, the number of waveguides varies between two and ten. For the waveguide thickness we choose the maximum value of 200 nm, which is determined by the maximum tolerable film stress for the deposition process of Si_3_N_4_. The 200 nm thick waveguides are multimode.

Using a minimum bend radius of 300 μm for low bend loss and minimizing overlap with the excitation waveguides for low cross talk, the waveguides are routed towards a chip edge, where these are merged into a single waveguide with a width of 500 μm. The latter waveguide is tapered down to 105 μm towards the chip edge for optimum coupling to a multimode fiber (a Thorlabs FG105UCA fiber, core diameter 105 μm). The fiber output can be coupled to a spectrometer.

In this work, we concentrate on optical trapping and Raman excitation using the excitation waveguides, while the Raman signals are collected with an objective (see the Experimental section). Actual use of the detection waveguides is left for future work. Their design is reported here for completeness.

### The microbath

The microbath is a cylindrical volume (compare Figure 4b) to be filled with sample fluid. Among the devices, the diameter of the cylinder is in the range of 5–60 µm. For the 2-waveguide device, the microbath is shaped as a linear channel with a rectangular cross section (Figure 4a). For the microbath we face two issues, namely the entrapment of air bubbles during filling and the quick evaporation of the small volume of sample fluid before use. The first issue is overcome by adding two side channels to the microbath, enabling filling from the end of one of these. For this purpose, one channel is designed wider near its end, using a funnel shape (see Figure 6a below). The wide side of the funnel measures 250 μm across, a size that is suitable for the thin needle of a syringe. When a droplet is applied to the funnel, capillary forces induce rapid progress of the fluid/air interface towards the microbath. The pinning of the fluid/air interface at sharp edges [11] between the side channel and the microbath is avoided by designing smoothly curved walls at the transition. Thus, the microbath can be reliably filled completely, followed by filling of the other side arm. The filling process can be monitored with a microscope. The second issue is solved by building a macrobath on top of the microbath using an image spacer to enable a significant increase of the volume of sample fluid. See subsection on fabrication below.

## Experimental

### Fabrication of the multi-waveguide devices

**Figure 5:**
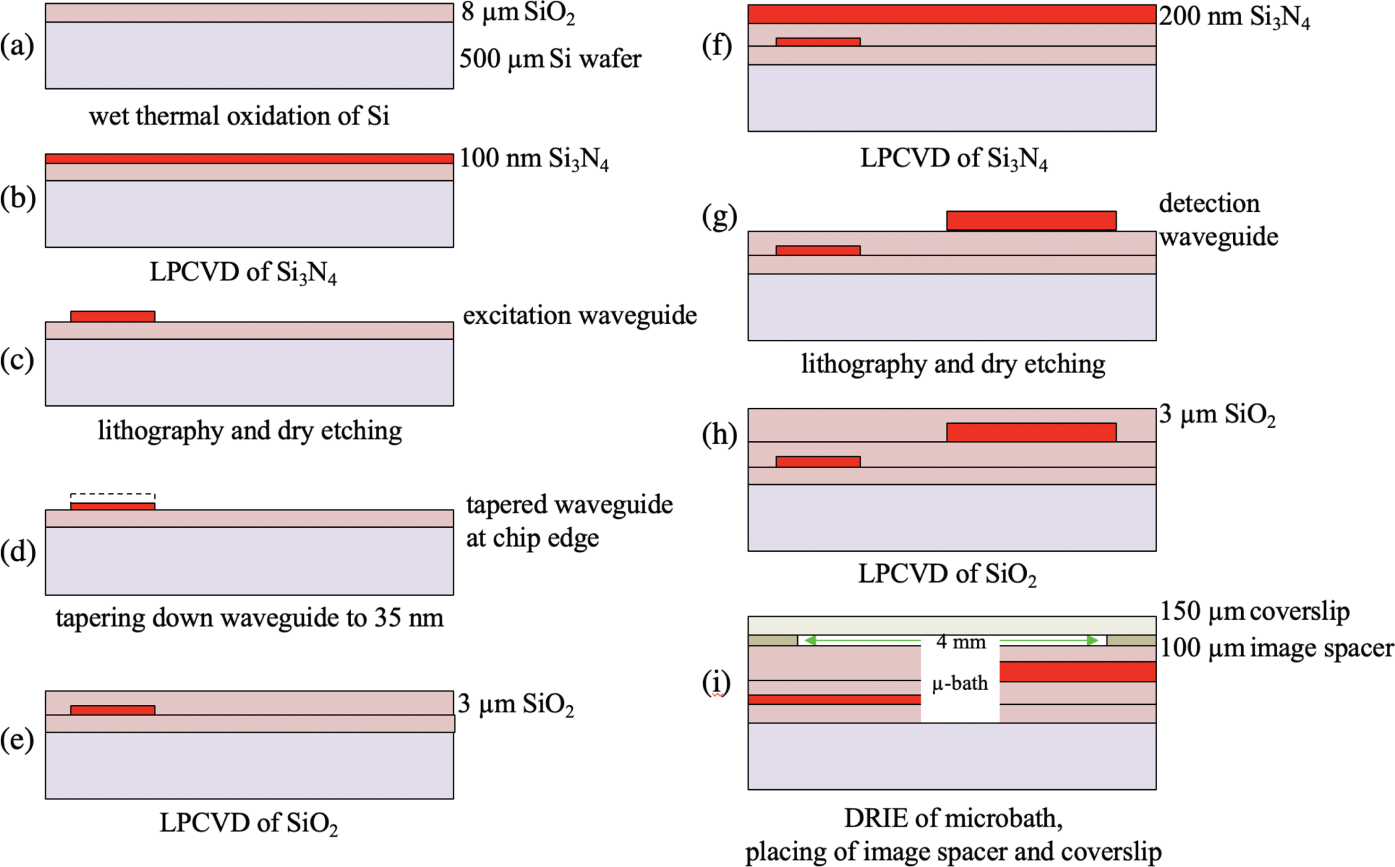
Main steps of the fabrication process of the multi-waveguide trapping and Raman devices based on Si_3_N_4_ waveguides. Under each cross section the step is mentioned. The cross section of step d) is at the chip edge, where the waveguide reaches a thickness of 35 nm as a result of the tapering down. For reference, the original waveguide thickness of 100 nm is indicated in d) as well (dashed part). In step i), the side channels of the microbath, etched using the same deep reactive ion etching (DRIE) procedure, have been omitted. The thickness of the various layers does not reflect the real situation. The surface topography resulting from the conformal deposition on the etched structures has been omitted in the cross sections.

The devices were fabricated based on the designs and the simulations described in the preceding section. In Figure 5, we show the main fabrication steps, which are performed on a batch of 100 mm silicon wafers. The overall design comprises 30 chips of size 11 mm × 11 mm. 28 chips each have a single trapping/Raman device, with up to 32 excitation and up to 10 detection waveguides. The remaining chips have control structures.

The first fabrication step is the wet thermal oxidation of Si at 1150 °C to obtain an 8 µm thick layer of SiO_2_ (Figure 5a). This layer serves as the bottom cladding for the excitation waveguides. Its thickness is chosen such that the 785 nm light within the excitation waveguides is completely decoupled from the silicon substrate.

**Figure 6:**
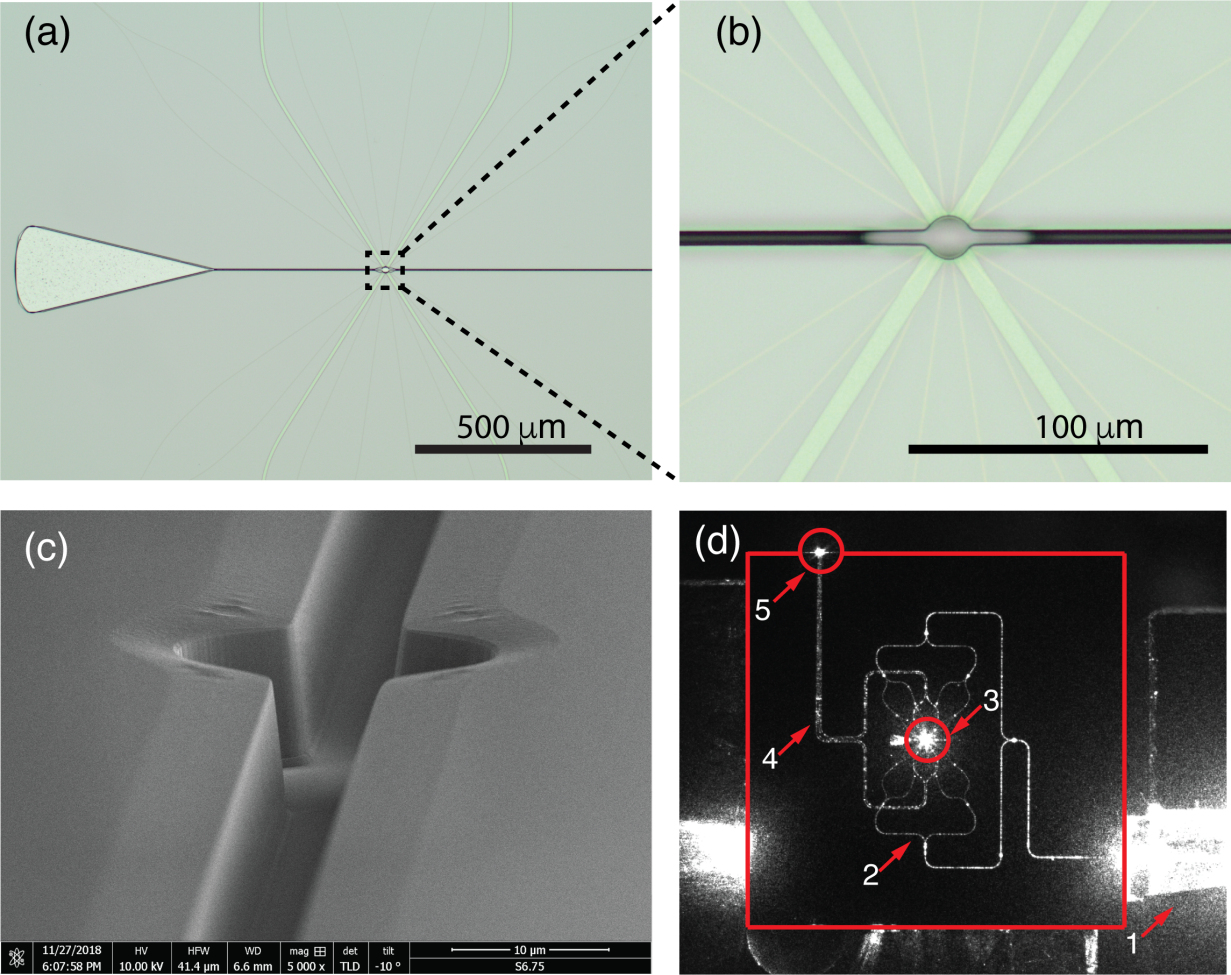
(a) Optical microscope image of a device with 16 excitation and 4 detection waveguides. (b) Magnification of the marked area in (a), clearly showing the 16 excitation waveguides and the 4 detection waveguides. (c) Scanning electron microscope image of the device, showing the 15 μm diameter central microbath with the 5 μm wide side channels. (d) Camera image of the 16-waveguide device actuated with light from the input fiber, which is embedded in a fiber array unit (FAU). The various structures light up as a result of light scattering, giving bright saturation of the camera. The red square indicates the chip edges. 1: FAU. The large saturation region results from scattering loss at the input waveguide. 2: Excitation-waveguide circuitry. 3: Microbath with the central trapping region. 4: Detection-waveguide circuitry. 5: Bright spot resulting from scattering of 785 nm light, coupled out from the multimode waveguide connected to the detection waveguides. The detection waveguides collect this light from the microbath at their facets, as a result of direct illumination and scattering.

Then, a 100 nm thick layer of Si_3_N_4_ is deposited using low pressure chemical vapor deposition (LPCVD, Figure 5b). This layer is patterned using optical lithography and reactive ion etching (RIE) in a fluorine-based plasma, which is followed by resist stripping (Figure 5c). The resulting 1 µm wide excitation waveguides and the related circuitry have low propagation loss (≈ −0.5 dB·cm^−1^ for the straight sections). One waveguide (the input waveguide) starts at the chip edge and is split into *N* waveguides using (*N* − 1) 50/50 Y-splitters. The *N* waveguides are routed to point radially towards the position that becomes the device center, similar to the example in Figure 4b. For multiple waveguides (*N* > 2), the overall waveguide circuitry resembles a flower as can be seen in Figure 6d below.

In this stage, the input waveguide is adiabatically tapered down along a length of 1000 µm to a thickness of 35 nm at the chip edge for optimum fiber-to-waveguide coupling using a special tapering procedure. This step is illustrated in Figure 5d. Here, the solid part of the waveguide is 35 nm thick, while the dashed part indicates its regular 100 nm thickness away from the edge.

In the next step, a 3 µm thick layer of SiO_2_ is deposited using LPCVD (Figure 5e). This layer acts as an upper cladding of the excitation waveguides and separates these from the waveguiding layer that follows. For simplicity, we do not show the surface topography resulting after LPCVD due to waveguides already present.

Subsequently, 200 nm of Si_3_N_4_ is deposited using the same LPCVD process as for the excitation waveguides (Figure 5f). Using lithography, RIE and resist stripping, we produce multiple detection waveguides in this layer fanning out from the central device region (Figure 5g). The detection waveguides are routed away from the center as a waveguide array and at the chip edge are merged into a multimode waveguide suitable for coupling to a multimode fiber. A thickness of 200 nm is appropriate for the Si_3_N_4_ layer, since it is just below the critical thickness that results in layer cracking due to stress after deposition.

Then, a 3 µm thick layer of SiO_2_ is deposited by LPCVD, which acts as the top cladding for the detection waveguides and as a protection layer (Figure 5h).

The final in-line step is the etching of the cylindrical microbath centered at each chip (compare Figure 4b) using deep reactive ion etching (DRIE). This is a critical step, since the etch goes 14.3 µm deep down to the substrate, through all the device layers, including the waveguide circuitry at two levels. The etch is highly anisotropic and produces smooth walls of the microbath and thus smooth waveguide facets. For this step, we use a double layer resist (hard mask/photoresist) for good dimensional control and high etch resistance. For most devices, to facilitate filling, the microbath has side channels (see subsection on the microbath), which are etched simultaneously with the microbath. After dicing of the wafer, a millimeter-scale macrobath is created on each chip by placing a 100 µm thick imaging spacer (Biolink Relink 1300) with a 4.0 mm hole. The adhesion strength of the top and bottom surface of the image spacer are different. The weaker adhesive is affixed to the chip to enable its residue-free removal, facilitating device reusability. The stronger adhesive is used to seal the macrobath, which serves as supply volume for the microbath. The sample fluid is then introduced such that after filling a convex meniscus bulges out above the macrobath. Finally, the sample volume is sealed with a 150 µm thick coverslip by pushing it onto the sticky imaging spacer, thus reaching the stage shown in Figure 5i. Owing to the meniscus, fluid evaporation is not fast enough to cause air inclusion under the coverslip during sealing.

In Figure 6, we give an impression of the final fabrication result for a 16-waveguide device. The device overview is presented in Figure 6a, where the microbath, the side channels, the funnel and the four detection waveguides can be seen. Figure 6b shows the magnified area indicated by dashed lines in Figure 6a. Here, the 16 narrow excitation waveguides are also clearly discernible. The four detection waveguides occupy a maximum space along the sides of the microbath for optimum collection efficiency. The scanning electron microscope (SEM) image in Figure 6c shows the topography of the microbath and the side channels. The DRIE process of these structures is seen to be highly anisotropic, while giving smooth sidewalls. The facets of the excitation and detection waveguides are part of the cylindrical walls of the microbath and cannot be seen here. The surface adjacent to the microbath and the side channels is slightly angled. This feature occurs, because this device is made from a dummy wafer, for which we only used photoresist as the masking layer during the DRIE process. The actual devices used for the experiments do not have this feature, since in their fabrication, we applied the double layer resist described above. Figure 6d is a camera image of the 16-waveguide device actuated by 785 nm laser light. As a result of light scattering, both the excitation and the detection waveguides light up. A further indication of the operation of the detection waveguide is the bright spot at the chip edge indicated with the number 5.

### Experimental setup and sample preparation

The experimental setup is based on a Sacher 785 nm laser (Sacher TEC-420). From the primary laser beam, two beams are formed using a 50/50 beam splitter. Each beam is coupled into a single-mode polarisation maintaining fiber. The first of these fibers is butt-coupled to the input waveguide of the chip using a fiber array unit (FAU) glued to its end. The chip is mounted on a sample holder. The FAU is aligned using manual translation stages for coarse alignment and piezoelectric stages for fine alignment. The polarization of the light coupled out by the fiber is perpendicular to the plane of the chip. We optimize the fiber-to-waveguide coupling by maximizing the lighting up of the waveguide circuitry. This lighting up results from scattering of the propagating waveguide modes and can be monitored using an air objective and a camera mountable above the chip.

The light coupled out by the second fiber is collimated to a free space beam and expanded to properly overfill the objective (Olympus water immersion objective, 60×/1.2 NA) of a home-built LTRS setup. This objective can be placed just above the coverslip on the chip. Using the tweezers functionality of the LTRS, a particle (we use polystyrene beads; see below) can be grabbed from the fluidic volume, transported and then be handed over to a multi-waveguide trap. This transport is actually carried out by translating the main stage, on which the assembly with the sample holder and the small stages are mounted, while the LTRS-trapped particle remains in position. This procedure is very effective in supplying a multi-waveguide trap with a particle. The Raman branch of the LTRS is used for measuring the Raman spectrum of the particle trapped by a multi-waveguide trap. The Raman spectrum is generated by the same on-chip beams that induce the trapping of the bead. Using shutters, we can quickly switch between the tweezers functionality and the chip functionality.

The particles we use for trapping and Raman spectroscopy are polystyrene beads (Nanosphere, ThermoFisher) with diameters of 1 and 3 μm. We prepare bead suspensions with a concentration of about 10^6^ mL^–1^. The suspensions are sonicated to obtain a homogeneous bead distribution. The chip is then filled with the sample fluid, as described above in relation to Figure 5i, and is then closed with a coverslip.

## Results and Discussion

### Optical trapping with a 2-waveguide trap and a 16-waveguide trap

After transporting a bead with the LTRS to a position near the center of a multi-waveguide device, we release the bead and simultaneously actuate the multi-waveguide device. The bead then almost immediately snaps into a near trapping site in the microbath. When the power offered by the fiber, *P*_fib_, to the chip’s input waveguide is high enough, the bead can remain stably trapped in the local trap for tens of minutes. For a 3 μm bead in the 16-waveguide device, this snapping into a local trap is demonstrated in the video of Supporting Information File 1. In both the 2-waveguide and 16-waveguide device, there are multiple local traps where a bead can be stably trapped, as demonstrated for a 3 μm bead in the 2-waveguide device in Supporting Information File 2. Even more, multiple beads can be stably trapped simultaneously in different local traps, as demonstrated for two 1 μm beads in the 16-waveguide device in Supporting Information File 3. We observe trapping events of a 1 μm bead in the 2-waveguide device for *P*_fib_ = 8 mW, while in the 16-waveguide device, we already observe trapping for *P*_fib_ = 1 mW. For such low powers the bead can hop between local traps. This hopping is visible by eye in the camera image and can be seen for a 3 μm bead in the 2-waveguide device in Supporting Information File 4. With increasing *P*_fib_ the local traps becomes stronger, resulting in stronger confinement of the Brownian motion of a bead in the local trapping potential.

For the quantitative characterization of the 2- and 16-waveguide traps, we study the confined Brownian motion of single trapped beads by recording videos, using a high-speed CMOS camera (AV Mako U029, pixel size 4.8 μm) and by tracking the bead position as a function of the time in these videos. Each video typically contains about 9000 frames, taken at a frame rate of 541 fps and an exposure time of 1 ms. The videos are recorded for ten values of *P*_fib_. We track the bead positions using a template matching algorithm [12]. Briefly, we calculate the 2D cross-correlation between an example image of only the bead (taken from one of the frames) and each frame of the video. The maximum in the respective correlation maps indicates the position of the bead. We find the position of the maxima with a resolution of a few nanometers by fitting a 2D parabola to the correlation maps in a limited range near the maximum.

**Figure 7:**
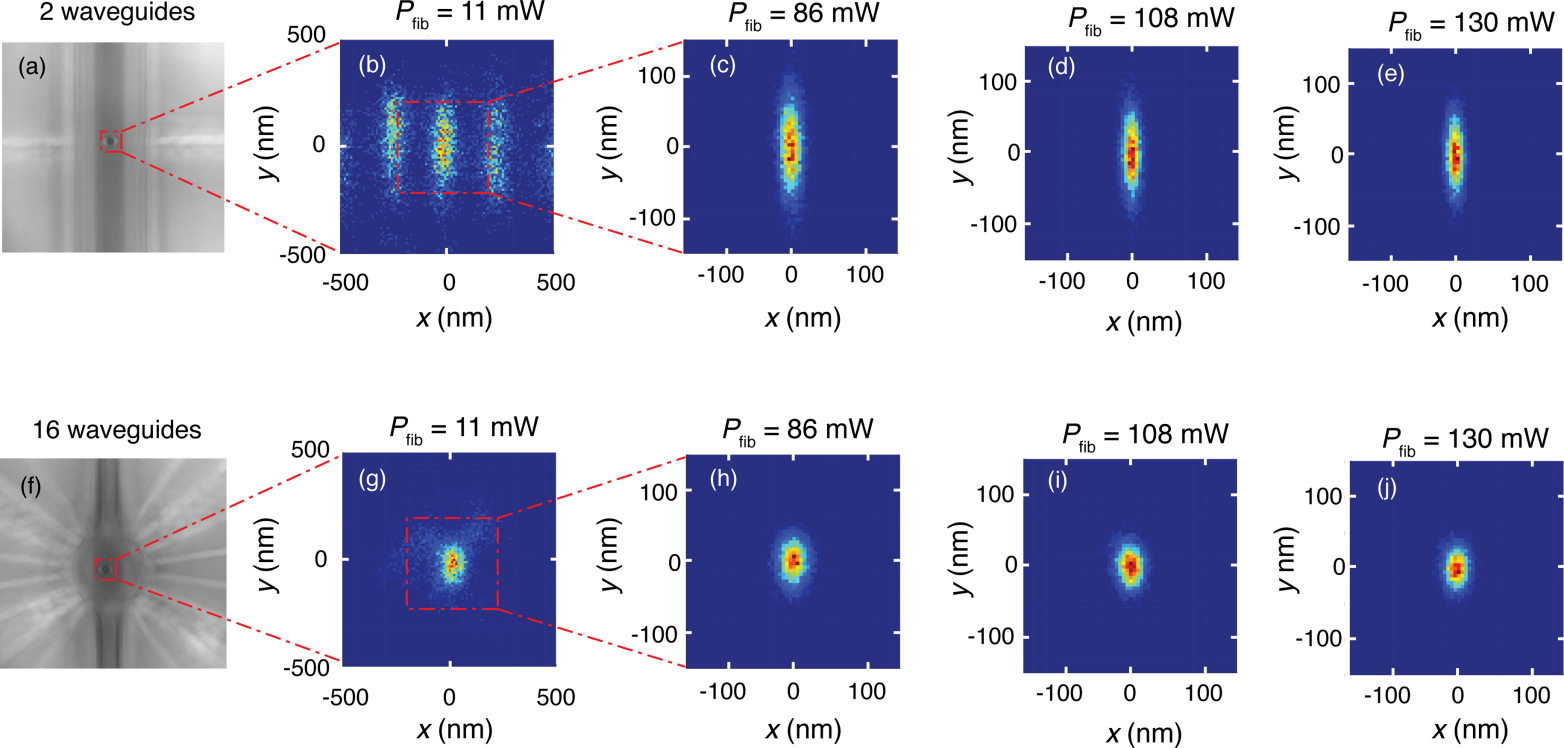
(a) and (f) are optical microscope images of the 2-waveguide and 16-waveguide trap, respectively, with a trapped 1 μm polystyrene bead in the center. Further images in the upper (lower) row are histograms of the position of the 1 μm bead in the 2 (16)-waveguide trap, for increasing values of *P*_fib_.

In Figure 7, examples of 2D histograms of the position of a 1 μm bead obtained from template matching are presented for the 2-waveguide trap (upper row) and the 16-waveguide trap (lower row) and for various values of *P*_fib_. The left side of the figure shows microscope images of the central part of each device, with the region indicated where the position tracking has been performed. For both traps, the bead is delivered by the laser tweezers close to the central trapping site of the chip.

For the 2-waveguide trap and *P*_fib_ = 11 mW, the histogram closely resembles part of the interference pattern of Figure 4a. Thus, the bead is not localized in a single local trap, but hops by thermal stimulation between adjacent local traps. With increasing optical power, the motion of the bead becomes confined to the central local trap, while the excursions from its center become smaller, as seen in Figure 7c–e. This indicates that the trap becomes stronger. The excursions in the *y*-direction exceed those in the *x*-direction. This corresponds with the shape of the hot spots of the energy density in the 2-waveguide trap shown in Figure 4a. For the 16-waveguide trap, the histograms indicate stable trapping in the central local trap. Again, the bead excursions decrease with increasing power. Also in this case, the bead excursions in the *y*-direction exceed those in the *x*-direction, but the difference is smaller than for the 2-waveguide trap, in agreement with the shape of the central hotspot shown in Figure 4b.

Assuming that the traps are harmonic and using the equipartition theorem [13], we may write 
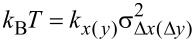
. Here *k_x_*_(_*_y_*_)_ is the trap stiffness for the in-plane directions, σ_Δ_*_x_*_(Δ_*_y_*_)_ is the standard deviation of the Gaussian curve describing the 1D histogram reflecting the bead position for these directions, *k*_B_ is the Boltzmann constant and *T* = 293 K is the temperature. Generally, the proportionalities *k_x_*_(_*_y_*_)_ ∝ *P*_trap_ and 
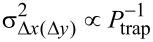
 are found to hold (*P*_trap_ is the power offered to the trap by the waveguides), in agreement with the above relation. By fitting 2D Gaussian functions to the 2D histograms, we obtain σ_Δ_*_x_*_(Δ_*_y_*_)_ and, thus, *k_x_*_(_*_y_*_)_ as a function of *P*_fib_. In this procedure it is not needed to take into account the small correction of σ_Δ_*_x_*_(Δ_*_y_*_)_ due to motion blurring [13], in view of the short integration time of the camera compared to the trap relaxation time.

**Figure 8:**
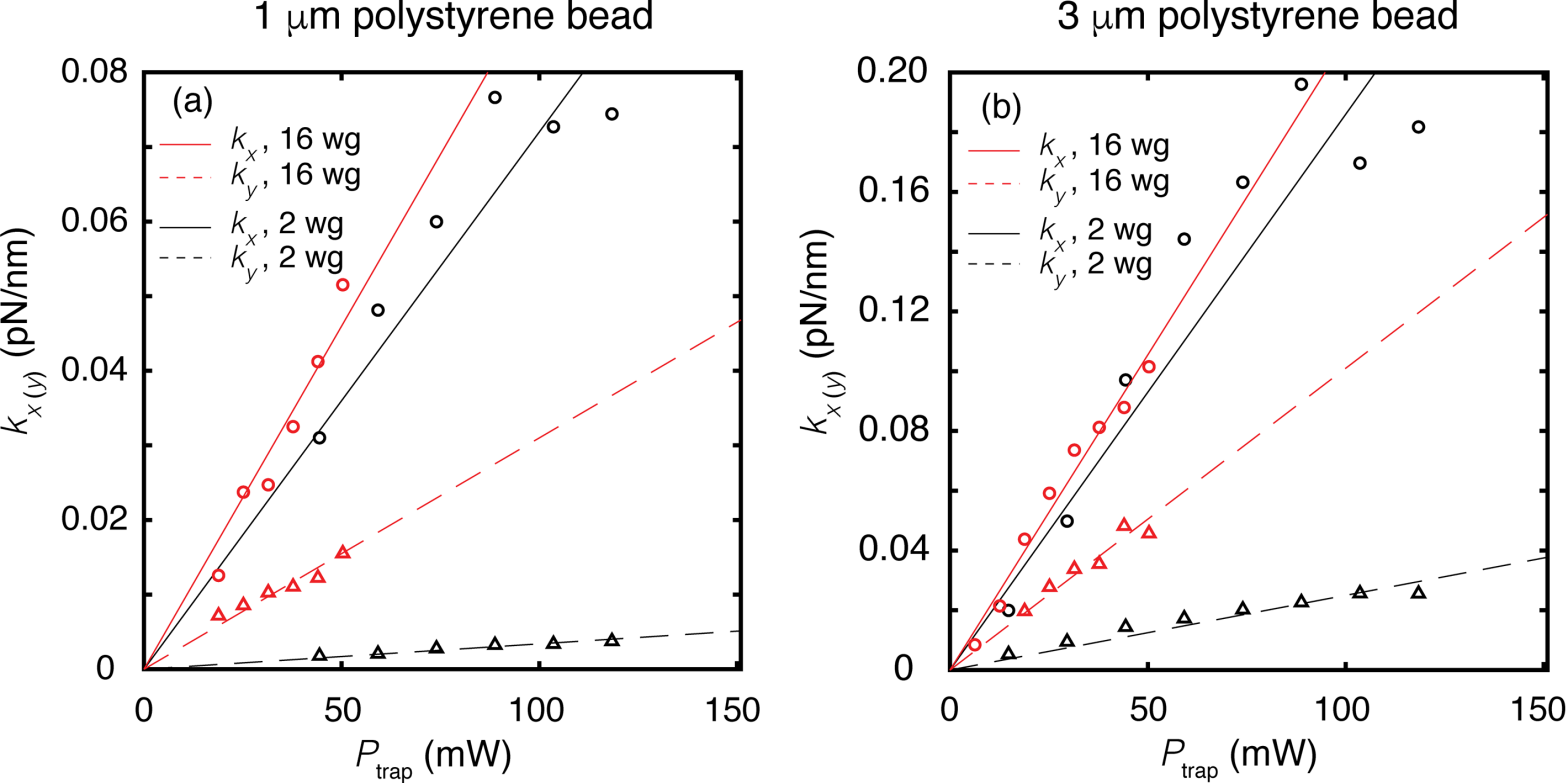
Trap stiffnesses *k_x_*_(_*_y_*_)_ as a function of the power *P*_trap_, represented by empty circles (triangles) and solid (dashed) lines, for the 2-waveguide (black) and 16-waveguide (red) traps, for trapped polystyrene beads of 1 and 3 μm diameter. The lines are linear fits to the data points.

To obtain plots of *k_x_*_(_*_y_*_)_ as a function of *P*_trap_, we need to convert *P*_fib_ to *P*_trap_. For the conversion we use the following approach, taking into account that the 2-waveguide trap has one splitter and the 16-waveguide trap has 15 splitters, arranged in four stages. For the same *P*_fib_ offered to the input waveguide, the expected fiber-to-waveguide loss of −0.5 dB and the loss at each Y-splitter of −0.5 dB (see subsection on fabrication) lead to an estimated ratio of the power offered to the 16-waveguide and the 2-waveguide trap of *P*_trap,16_/*P*_trap,2_ ≈ 0.7. From measurements of the power coupled out vertically from the microbath of either trap due to light scattering in the absence of beads, a power expected to be proportional to *P*_trap_, we obtain about 0.4 for this ratio. With some bias, we attribute the factor of about 0.6 between the estimated and the measured ratio to the suboptimal fiber-to-chip coupling and other additional losses for the 16-waveguide trap. Thus, we know all transmission factors needed for the conversion of *P*_fib_ to *P*_trap,2_ and *P*_trap,16_ and can obtain the plots of *k_x_*_(_*_y_*_)_ versus *P*_trap_, as shown in Figure 8. We have limited the number of data points to those *P*_trap_ values for which the bead is stably trapped in the central local trap of the 2-waveguide trap, i.e., for which hopping between local traps does not occur. Further, in the plots, the maximum value of *P*_trap,16_ is lower than the maximum value of *P*_trap,2_ as a result of the different conversion factors between *P*_fib_ and *P*_trap_ of the traps. The plots also show linear fits to the data points. On average, the fits describe the data points rather well, although for the 2-waveguide trap, the scatter of the data points *k_x_*(*P*_trap_) is stronger than for the other data points. The finding of linearity agrees with the proportionality *k_x_*_(_*_y_*_)_ ∝ *P*_trap_.

The slope of the fits in Figure 8 yields the normalized experimental trap stiffness *k_x_*_(_*_y_*_),exp,_*_n_* (unit: pN·nm^−1^·W^−1^), a quantity suitable for comparison. The resulting values of *k_x_*_(_*_y_*_),exp,_*_n_* are compiled in Table 1, along with the values of *k_x_*_(_*_y_*_),sim,_*_n_* obtained from the force–distance relations derived from the FDTD simulations of the type we report in [6].

**Table 1:** Normalized experimental and simulated values of the trap stiffness, *k_x_*_(_*_y_*_),exp,_*_n_* and *k_x_*_(_*_y_*_),sim,_*_n_*, respectively, for the 2-waveguide and 16-waveguide trap and for trapped 1 and 3 µm polystyrene beads.

Number of waveguides	Bead diameter (μm)	*k_x_*_,exp,_*_n_*	*k_x_*_,sim,_*_n_*	*k_y_*_,exp,_*_n_*	*k_y_*_,sim,_*_n_*
		(pN·nm^−1^·W^−1^)	(pN·nm^−1^·W^−1^)	(pN·nm^−1^·W^−1^)	(pN·nm^−1^·W^−1^)

2	1	0.72	0.50	0.034	0.024
	3	1.86	1.65	0.25	0.58

16	1	0.92	1.79	0.31	0.17
	3	2.11	2.31	1.01	1.32

The experimental stiffness values in Table 1 confirm that the 2-waveguide trap is stiffer in the *x*-direction than in the *y*-direction. The same holds for the 16-waveguide trap, but the effect is smaller, as already observed visually from the histograms in Figure 7. Moreover, the experimental stiffness values of the 16-waveguide trap systematically exceed the corresponding values of the 2-waveguide trap, convincingly confirming stronger light concentration in the former case as result of the interference of the 16 beams. The stiffness values for the 3 μm bead exceed the corresponding ones of the 1 μm bead, since a larger volume is subject to the energy density of the optical field, leading to a higher force.

In more detail, defining 
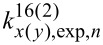
 as the normalized experimental stiffness of the 16(2)-waveguide trap, it follows from Table 1 that the ratios 
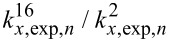
 and 
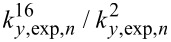
 for the 3 μm bead are smaller than these ratios for the 1 μm bead. This difference of the ratios results from the different character of the energy-density distributions of the 2- and 16-waveguide trap probed by the beads trapped in the center of these traps. The energy-density distributions are characterized by hot stripes (2-waveguide trap) and hot spots and hot partial rings (16-waveguide trap), all typically 295 nm apart (see Figure 4). Going from 1 μm to the 3 μm bead size in the 2-waveguide trap, the bead probes more hot stripes of equal intensity (see Figure 4). For the 16-waveguide trap, on the contrary, the bead-size increase leads to probing of more hot partial rings of lower intensity than that of the three central hot spots (see Figure 4). This results in a smaller increase of the optical force than for the 2-waveguide trap. The simulated stiffness values are close to the experimental ones, with the average of the ratio *k_x_*_(_*_y_*_),exp,_*_n_*/*k_x_*_(_*_y_*_),exp,_*_n_* being 1.05, while the minimum and the maximum of this ratio are 0.43 and 1.82, respectively. We consider this to be in good agreement. Upon bead-size increase, the ratios 
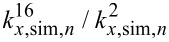
 and 
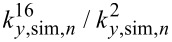
 show similar behavior as the above experimental counterparts, emphasizing agreement of the experimental and simulated results.

The above results for polystyrene beads are promising for extending the experiments to biological particles, which have a lower refractive index contrast with respect to water than polystyrene and are thus harder to trap. Trapping of polystyrene beads already starts for powers of several milliwatts. Thus, we have quite some power left for making the transition to stable trapping of, for example, bacteria, human cells or extracellular vesicles. The 16-waveguide device is the better choice in this respect, since Table 1 indicates that it clearly has a higher trap stiffness. Finally, the stiffness values of the 16-waveguide device are similar to those of tightly focused Gaussian beam traps, which are also used for trapping of polystyrene beads. See for example [14]. Since such Gaussian beam traps have also been used for trapping of a wide range of biological particles [2], this is a further indication that the 16-waveguide device can be used for this purpose as well.

### Raman spectroscopy with the 2-waveguide trap and the 16-waveguide trap

**Figure 9:**
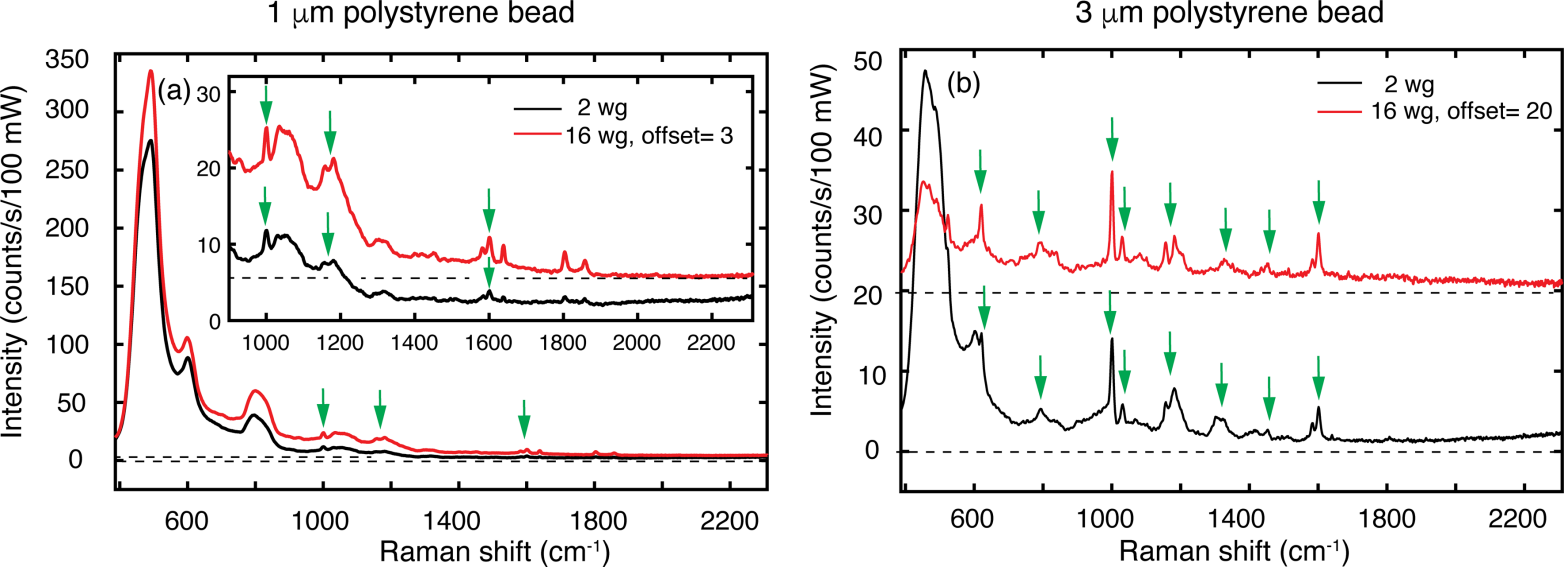
Raman spectra obtained with the 2-waveguide (black line) and the 16-waveguide (red line) trap for polystyrene beads with diameters of 1 and 3 μm. For the spectra obtained with the 16-waveguide trap, vertical offsets were applied as indicated in the legend. Each spectrum has its own horizontal axis drawn as a dashed black line. Identified characteristic Raman peaks of polystyrene are denoted by arrows. In (a) the inset is a magnification of a part of the main plot.

We recorded Raman spectra of trapped polystyrene beads, induced by the beams from the waveguides and collected from the top by the objective of the LTRS. Examples of smoothed Raman spectra (three point moving average) of single 1 and 3 μm beads for the two traps are shown in Figure 9. To enable a direct comparison of the peak heights, the spectra are normalized to the integration time of 60 s and to *P*_trap,2_ and *P*_trap,16_ obtained from *P*_fib_ = 157 mW. For the conversion of *P*_fib_ to *P*_trap,2_, we had to use a different factor than discussed previously, since in preparing for these Raman experiments, we deduced *P*_trap,16_/*P*_trap,2_ ≈ 1 from the powers coupled out vertically. We attribute the different conversion factor to a lower fiber-to-chip coupling for the 2-waveguide trap.

For either trap, the spectra for both bead sizes show distinct polystyrene Raman peaks [15] (in the figure indicated by arrows), although the spectra for the 3 μm bead are clearly richer. The Raman signals from the 16-waveguide trap are stronger than from the 2-waveguide trap, for both bead sizes. For example, the 1001 cm^−1^ peak for the 16-waveguide trap for the 1 and 3 μm bead has 76% and 22% more counts, respectively, than for the 2-waveguide trap. The higher percentage for the 1 μm bead than for the 3 μm bead also here results from the different character of the energy-density distributions probed by these beads in the center of the 2- and 16-waveguide trap, as discussed above in relation to the ratio of trap stiffness for the 2- and 16-waveguide trap.

For low wavenumbers, up to about 900 cm^−1^, the spectra show a strong background with peaks at 450, 590 and 800 cm^−1^. We find that the background also occurs for the empty trap, but then it is much weaker. This indicates that its strength results from scattering of light at the trapped particle towards the objective. Thus, the light leaving the waveguides is the source of the background, implying that the background is generated in the waveguide circuitry. The background is much higher for the 1 μm bead than for the 3 μm bead. We explain this in relation to the Raman collection volume, which has an in-plane diameter of 1 μm, as determined by the 40 μm diameter confocal pinhole. For the 3 μm bead, the Raman collection volume is mainly inside the bead. Therefore, most of the light scattered at the bead surface is not collected by the objective. For the 1 μm bead, the Raman collection volume includes the bead surface, thus leading to a higher contribution to the background. In our future work, we will develop a background-subtraction procedure to recover the Raman signals that are obscured now by the background. We note that background signals of various shapes generated in Si_3_N_4_ waveguides are also observed by other groups in waveguide Raman spectroscopy [16–17].

## Conclusion

We have presented the design, fabrication and performance demonstration of multi-waveguide devices for on-chip optical trapping and Raman spectroscopy of particles in a fluidic environment. The new concept we implement in these integrated photonics devices is that launching of multiple beams (>2) from various directions towards the center of the microbath leads to a strong field enhancement in the center and considerably counteracts the unwanted effect of light concentration near the waveguide facets. Thus, a region of preferential trapping is realized around the device center, where several hot spots resulting from interference act as traps for particles in the suspension. Guided by FDTD simulations, we arrive at the proper nanometer-scale thickness for the Si_3_N_4_ excitation waveguides to serve for trapping and Raman generation. FDTD simulations also lead to optimum arrangements of the waveguides around the microbath. Important realized features of these waveguides are optimum thickness for obtaining narrow and weakly diverging beams, small propagation losses, and optimum fiber-to-waveguide coupling for introducing light into the device by tapering down the thickness of the input waveguide near the chip edge. Microfluidic considerations lead to the design of a microbath with side channels for filling with a sample suspension, a process aided by capillary forces.

Experiments with two example devices, with 2 and 16 excitation waveguides, show clear trapping events for polystyrene beads of 1 and 3 μm diameter and confirm the existence of a configuration of hot spots for preferential trapping near the center of the 15 μm diameter microbath of the 16-waveguide device. Further features are hopping of the bead between adjacent local traps at low optical power and confined Brownian motion at higher power. A detailed study of the confined Brownian motion by tracking the position of the trapped beads in time yields the normalized trap stiffness. In particular, for the 16-waveguide device, the stiffness value is comparable to the values known for tightly focused Gaussian beam traps, which have been used already for trapping of biological microparticles. The experimental values of the normalized stiffness of the 16-waveguide trap are clearly higher than for the 2-waveguide trap.

Raman spectra of the trapped beads, induced by the multiple beams also used for trapping, show clear Raman peaks of polystyrene in spite of a pronounced background present in the low wavenumber range. We argue that the background is already present in the beams emitted by the waveguides and is thus generated in the waveguide material. The different strength of the background found for the two bead sizes suggests that the strength of the measured background results from the interplay of the particle size and the degree of confocal filtering.

The successful optical trapping of polystyrene microparticles and the Raman spectra are promising results, opening up possibilities for on-chip trapping and Raman spectroscopy of biological particles such as bacteria. Making this transition can be challenging, due to the lower refractive index contrast of biological particles with respect to water (thus making these particles harder to trap) and the lower concentration of molecules contributing to Raman peaks.

## Supporting Information

In the supporting information files we present videos that demonstrate the delivery of beads by the laser tweezers to the 2-waveguide trap and the 16-waveguide trap and optical trapping of the beads by these chip traps.

File 1 Delivery by the laser tweezers of a 3 μm polystyrene bead to the 16-waveguide device.

File 2 Stable trapping of a 3 μm polystyrene bead in different local traps of the 2-waveguide device.

File 3 Simultaneous trapping of two 1 μm polystyrene beads in different local traps of the 16-waveguide device.

File 4 Hopping of a 3 μm polystyrene bead between local traps in the 2-waveguide device.
